# Influence of tensile-strain-induced oxygen deficiency on metal-insulator transitions in NdNiO_3−δ_ epitaxial thin films

**DOI:** 10.1038/s41598-017-04884-2

**Published:** 2017-07-05

**Authors:** Seungyang Heo, Chadol Oh, Junwoo Son, Hyun Myung Jang

**Affiliations:** 1 Division of Advanced Materials Science (AMS), Pohang University of Science and Technology (POSTECH), Pohang, 790-784 Republic of Korea; 2 Department of Materials Science and Engineering (MSE), Pohang University of Science and Technology (POSTECH), Pohang, 790-784 Republic of Korea

## Abstract

We report direct evidence that oxygen vacancies affect the structural and electrical parameters in tensile-strained NdNiO_3−δ_ epitaxial thin films by elaborately adjusting the amount of oxygen deficiency (δ) with changing growth temperature *T*_D_. The modulation in tensile strain and *T*_D_ tended to increase oxygen deficiency (δ) in NdNiO_3−δ_ thin films; this process relieves tensile strain of the thin film by oxygen vacancy incorporation. The oxygen deficiency is directly correlated with unit-cell volume and the metal-insulator transition temperature (*T*_MI_), i.e., resulting in the increase of both unit-cell volume and metal-insulator transition temperature as oxygen vacancies are incorporated. Our study suggests that the intrinsic defect sensitively influences both structural and electronic properties, and provides useful knobs for tailoring correlation-induced properties in complex oxides.

## Introduction

Correlated transition metal oxides with partially-filled *d* electrons undergo intriguing metal-insulator electronic phase transition (MIT)^1, 2^ as a result of electron interactions^3^. The capability to control those exotic phenomena with external stimuli is a major subject in correlated oxide heterostructure research due to their extreme sensitivity of those materials systems near metal-insulator phase boundary. For example, in these materials, strain and doping can cause small changes in the crystal structures or charge density, and these changes can shift the phase boundaries, and thereby lead to large change in the electrical and optical properties^4–6^. Among the diverse stimuli, oxygen vacancies are inevitable defects in perovskite oxides (ABO_3−δ_, where δ is the oxygen deficiency) during the synthesis of oxide thin films. The quantity of oxygen vacancies can be stabilized in the range of δ, and thus have a strong influence on the crystal and electronic structures. Because oxygen vacancies have a strong influence on the *d*-band filling, as well as structural changes, the ability to control the degree of oxygen deficiency may provide the opportunity to tailor the electrical^7^, optical^8^ or electrochemical device functionalities^9–11^ of ABO_3−δ_.

Bulk rare-earth nickelates (*R*NiO_3_, where *R* is a trivalent rare-earth ion) have strongly-correlated unpaired electrons in Ni^3+^ *e*_*g*_ band, and therefore undergo a first-order phase transition to an insulating state at 200 K due to strongly correlated unpaired electrons in Ni^3+^ *e*_*g*_ band under cooling^12^. Because nickel ions tend to be stabilized as Ni^2+^ valence state, instead of Ni^3+^, bulk *R*NiO_3_ is prone to oxygen deficiency unless it is synthesized under extremely high partial pressure *p*(O_2_) of oxygen^7, 13^. For this reason, the MIT and optical absorption coefficient of *R*NiO_3_ are both sensitively dependent on *δ* ^6, 7, 13–15^.

Likewise, *R*NiO_3_ epitaxial films synthesized using vacuum growth techniques (e.g., pulsed laser deposition^16^) are also prone to the oxygen deficiency due to the low *p*(O_2_) during the process. *R*NiO_3_ thin films show inconsistent MIT characteristics, and can have different *T*_MI_ even at the same lattice mismatch^6, 17–20^. For example, NdNiO_3_ (NNO) films grown on LaAlO_3_ substrates have *T*_MI_ ranging from 175 K to 0 K^6, 18, 20, 21^, which indicates the difficulty of synthesizing *R*NiO_3_ that has the desired cation ratio (*R*/Ni) and the degree of oxygen deficiency (δ). In the previous studies, oxygen deficiency in *R*NiO_3_ epitaxial films has been controlled by varying *p*(O_2_) during their growth^13, 15, 20^. However, *p*(O_2_) can significantly affect the cation stoichiometry of *R*NiO_3_ by increasing the scattering of ablated species in the background gas during pulsed laser deposition; the ablated elemental species experience different scattering conditions in different oxygen pressure as they propagates toward the substrate, influencing film cation stoichiometry, as well as oxygen deficiencies. Consequently, despite several studies that used *p*(O_2_) to modulate oxygen deficiency in *R*NiO_3_, to the best of our knowledge, no report has been demonstrated on the tuning of *T*_MI_ in *R*NiO_3_ using oxygen deficiency as a single variable while maintaining cation stoichiometry. To investigate the effect of pure oxygen vacancy on *T*_MI_ in *R*NiO_3_ thin films, their effect must be isolated from other stimuli such as cation stoichiometry and strain states.

In this Article, we report systematic control of oxygen deficiency in NNO epitaxial thin films with in-plane tensile strain and investigate the influence of oxygen deficiency on the MIT characteristics. In particular, unlike the previous reports, oxygen deficiency was spontaneously generated in tensile-strained NNO thin films, and was finely adjusted by varying growth temperature *T*_D_ without modifying other growth parameters; this result allows tuning the degree of oxygen deficiency while maintaining other stimuli. *T*_MI_ was directly correlated with the unit-cell’s structural expansion, which is a direct measure of oxygen deficiency. Consequently, these results demonstrate the influence of *T*_D_ on the formation of oxygen vacancies, and also provide insights into the effect of oxygen deficiency on the correlated phase in rare-earth nickelates.

## Results

**Figure 1:**
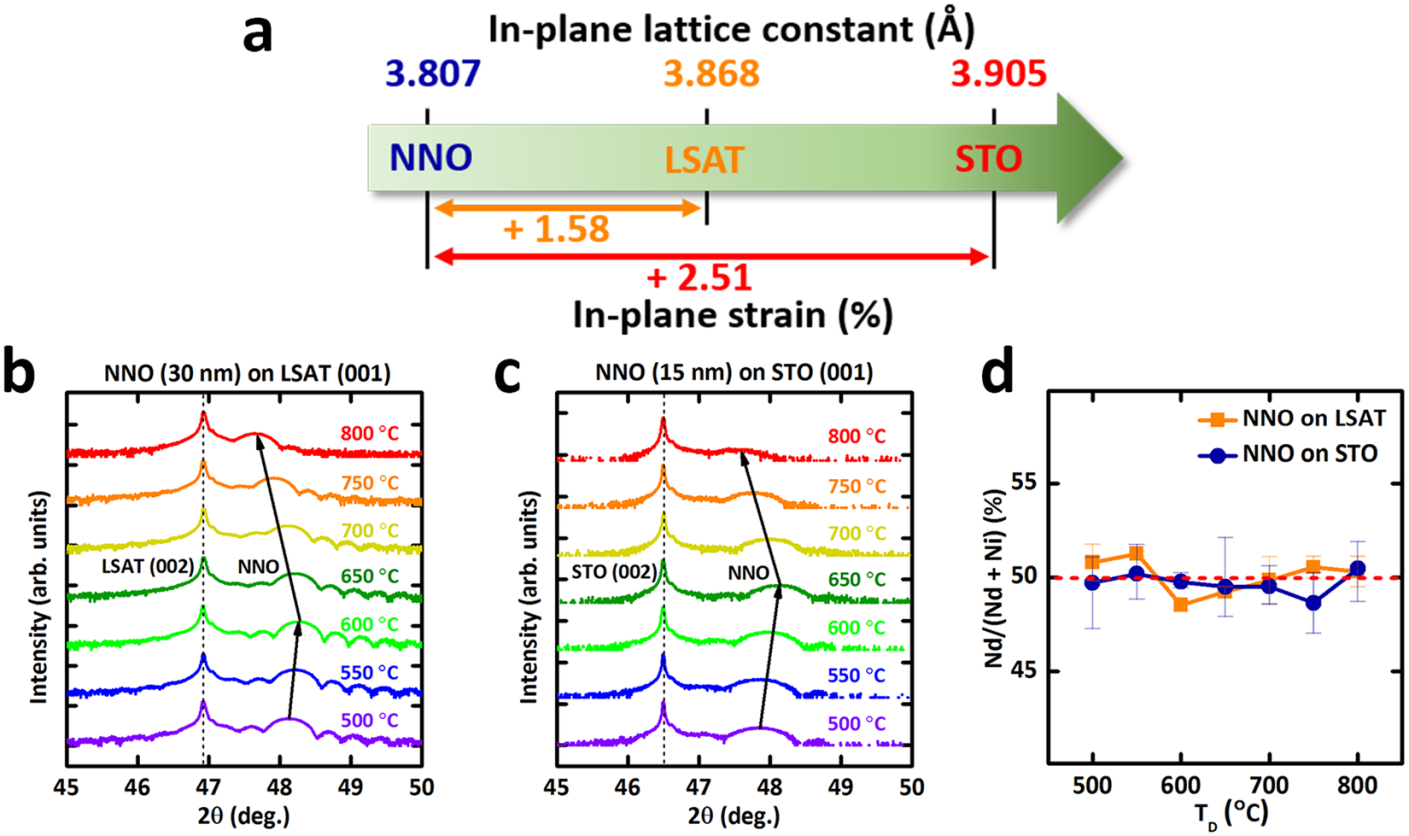
Tensile-strain-induced oxygen deficiency in NNO epitaxial thin films. (**a**) In-plane lattice constants and lattice mismatch of NNO with LSAT and STO substrates. (**b**,**c**) XRD θ–2θ scan of NNO epitaxial thin films on (001) LSAT (**b**) and (001) STO (**c**), as a function of growth temperature (*T*_D_). Black vertical lines: (002) diffraction peak of substrates (dashed) and pseudocubic NNO epitaxial thin films (solid). (**d**) Quantitative EDS analysis for cation stoichiometry in NNO thin films grown at various *T*_D_; cation atomic ratios of all samples differ by less than the error bar.

**Figure 2:**
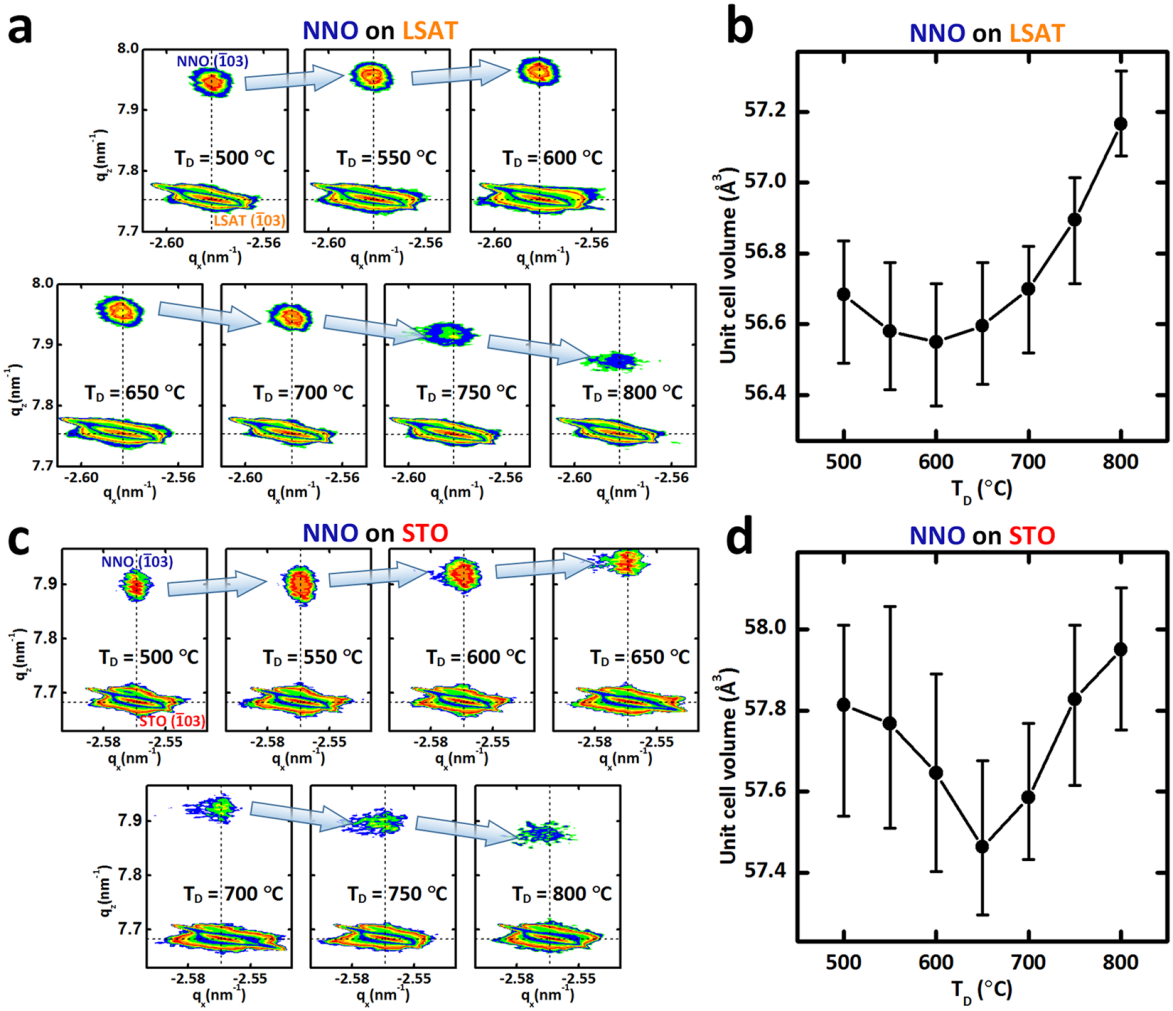
Structural modulation of tensile-strained NNO films as a function of oxygen deficiency. (**a**) Reciprocal space mapping (RSM) around the ($\bar{1}03$) LSAT Bragg peaks for 30-nm-thick NNO thin films grown at various *T*_D_. (**b**) Unit-cell volume of NNO epitaxial thin films grown at various *T*_D_ on LSAT substrate. (**c**) RSM around the ($\bar{1}03$) STO Bragg peaks for 15-nm-thick NNO thin films grown at various *T*_D_. (**d**) Unit-cell volume of NNO epitaxial thin films grown at various *T*_D_ on STO substrate.

Epitaxial NNO thin films were grown on (001)-oriented (LaAlO_3_)_0.3_-(SrAl_0.5_Ta_0.5_O_3_)_0.7_ (LSAT, *a* = 3.868 Å) or SrTiO_3_ (STO, *a* = 3.905 Å) single-crystal substrate by pulsed laser deposition at 500 ≤ *T*_D_ ≤ 800 °C under *p*(O_2_) = 300 mTorr with a KrF excimer laser fluence of 2.2 J/cm^2^. Increase in in-plane tensile strain tends to destabilize the Ni^3+^ valence state, and as a result, increase oxygen deficiency^22–24^; therefore, in-plane tensile strain was strategically applied in pseudocubic NNO films on both cubic substrates (+1.58% for NNO on LSAT and +2.51% for NNO on STO, Fig. 1a) to systematically generate oxygen deficiency. To ensure that NNO layers were fully-strained on both substrates (Fig. 2a,c), the NNO films used were 30 nm thick on LSAT and 15 nm thick on STO.

θ–2θ X-ray diffraction (XRD) symmetrical scan of NNO thin films on (001) LSAT (Fig. 1b) and STO (Fig. 1c) substrates grown at 500 ≤ *T*_D_ ≤ 800 °C show a (002) NNO peak near the (002) substrate peak; this observation indicates that the NNO films grew epitaxially in the c-axis orientation regardless of the growth temperature (*T*_D_). In both cases, as *T*_D_ increased from 500 °C, NNO peak was moved to slightly higher scattering angle until to 600 °C for NNO on LSAT, and until 650 °C on STO, then shifted to the lower scattering angle as *T*_D_ increased further. The decrease of the scattering angle represents the expansion of the out-of-plane lattice constant of the NNO thin films. To check the possibility of cation non-stoichiometry as an origin of this expansion, all samples grown at different temperature were characterized by energy dispersive spectroscopy (EDS). The cation atomic ratios of all samples were within 2% of 50% (Fig. 1d); this observation indicates that cation non-stoichiometry is unlikely to be the origin of the structural expansion. Instead, oxygen deficiency can be only modulated by *T*_D_, because the sticking coefficient and atomic mobility of light-weighted oxygen in the deposited film can be significantly influenced by *T*_D_ while the ablated elemental species (Nd, Ni) experience similar scattering conditions at the same laser fluence and *p*(O_2_). The presence of oxygen vacancies would modify the Ni valence from Ni^3+^ (r = 0.56 Å) to Ni^2+^ (r = 0.69 Å) by supplying electrons from the missing oxygen; this reduction process expands the crystal lattice by chemical expansion^25–27^.

To further verify structural modulation as a function of oxygen deficiency (δ), the volume change of the unit-cell was monitored using reciprocal space mapping (RSM) around the $(\bar{1}03)$ Bragg reflection of (001)-oriented NNO thin films grown at different *T*_D_ on LSAT (Fig. 2a) and STO (Fig. 2c) substrates. q_x_ was the same for all peaks from substrates and NNO films; this consistency means that all NNO thin films are fully-strained on both substrates (Fig. S1). The out-of-plane lattice constants extracted from RSM q_z_ values are consistent with those extracted from XRD θ–2θ scans (Fig. S1); this agreement confirms that the lattice expansion caused by oxygen deficiency is accommodated by constraint-free out-of-plane lattice expansion, whereas in-plane axes are tightly constrained by interaction with the substrate in both cases (Fig. 2a,c).

When the films are strained under tensile strain, the purely strain-induced out-of-plane lattice constant *c*_NNO_ of the NNO thin film can be accurately calculated as

1 $${c}_{{\rm{NNO}}}=[(1+\nu ){a}_{0}-2\nu {a}_{{\rm{NNO}}}]/[1-\nu ],$$

where ν = 0.35 (ref. 28) is Poisson’s ratio, *a*_0_ = 3.806 Å is the unstrained lattice constant, and *a*_NNO_ is the in-plane lattice constant of NNO (3.868 Å for NNO/LSAT (same as LSAT) or 3.905 Å for NNO/STO (same as STO)). Based on this relationship, estimated *c*_NNO_ under tensile strain were 3.741 Å on NNO/LSAT and 3.701 Å on NNO/STO. However, the smallest measured *c*_NNO_ were 3.768 Å on NNO/LSAT grown at 600 °C (i.e., 0.72% greater than calculated) and 3.778 Å on NNO/STO grown at 650 °C (i.e., 2.08% greater than calculated).

The calculations assume pure strain, so the observation that measured *c*_NNO_ exceeds calculated *c*_NNO_ indicates that, on both substrates, oxygen vacancies are incorporated into even optimized NNO films with the smallest unit-cell volume. Therefore, the experimental value of *c*_NNO_ cannot be simply understood by purely strain-induced Poisson-type contraction. Furthermore, the deviation from the pure strain effect (i.e., difference between measured *c*_NNO_ and calculated *c*_NNO_) becomes more pronounced in the case of more tensile-strained NNO films, i.e., NNO on STO. Previous researchers claimed that oxygen vacancies provide a channel that can relieve tensile strain in perovskite oxides grown on substrates with large lattice parameters; this claim suggests that the number of oxygen vacancies generated in NNO films to accommodate strain can increase as tensile strain increases^9, 22–24, 29^. Therefore, the deviation in unit-cell volume from that expected due to the pure strain effect is attributed to the formation of oxygen vacancies, which are increased by tensile strain.

In some perovskite oxides, e.g., CaMnO_3_, oxygen deficiency formed at high-temperature growth are unstable over time due to oxygen exchange with the environment, which is facilitated by tensile strain; when left exposed to ambient conditions, the films equilibrate and adopt a very small vacancy concentration without capping layer^30^. However, in our samples, the oxygen vacancies in NNO are not easily diffused out even under ambient conditions: To check time-dependent degree of oxygen deficiency in NNO films, we re-examined the change in the XRD peaks of all NdNiO_3−δ_ samples after 10 months (Fig. S2). Not only did all samples maintain their XRD pattern, but the trend of volcano shapes in out-of-plane scattering angles was also unchanged even after 10 months. Thus, although high degree of oxygen deficiency generated at high temperature are in a metastable state, oxygen exchange with atmosphere appeared to be suppressed in our NdNiO_3−δ_ films because of very limited kinetics of oxygen (or oxygen vacancies) at room temperature. Indeed, previous literature reported that the out-of-plane oxygen vacancy migration barrier of NNO is much larger than that of other perovskite oxide system, such as CaMnO_3_^31^.

**Figure 3:**
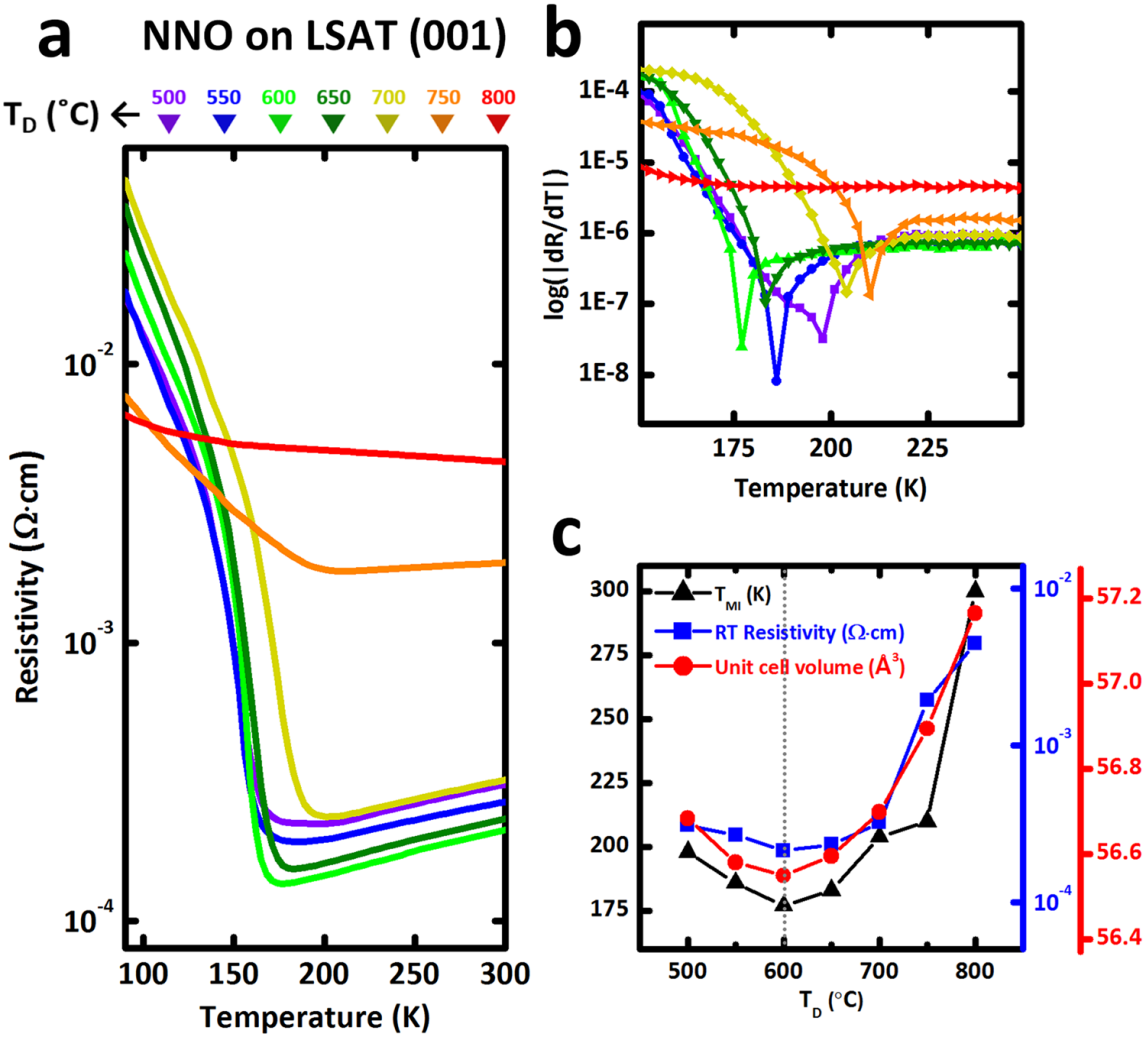
Correlation between MIT characteristics and structural modulation in oxygen-deficient NdNiO_3−δ_ films on LSAT substrates. (**a**) Temperature (*T*)-dependent in-plane resistivity for the NNO films on LSAT grown at various *T*_D_. (**b**) The first derivative of resistivity with respect to temperature (d*R*/d*T*) as a function of growth temperature (*T*_D_) to clarify *T*_MI_. (**c**) Correlation between *T*_MI_, room temperature (RT) resistivity and unit-cell volume in oxygen-deficient NdNiO_3−δ_ films. In case of NNO/LSAT grown at 800 °C, temperature dependence of resistivity curves show all insulating behaviors within the measured range (90 to 300 K), which indicates that *T*_MI_ is outside the plot window (*T*_MI_ > 300 K).

**Figure 4:**
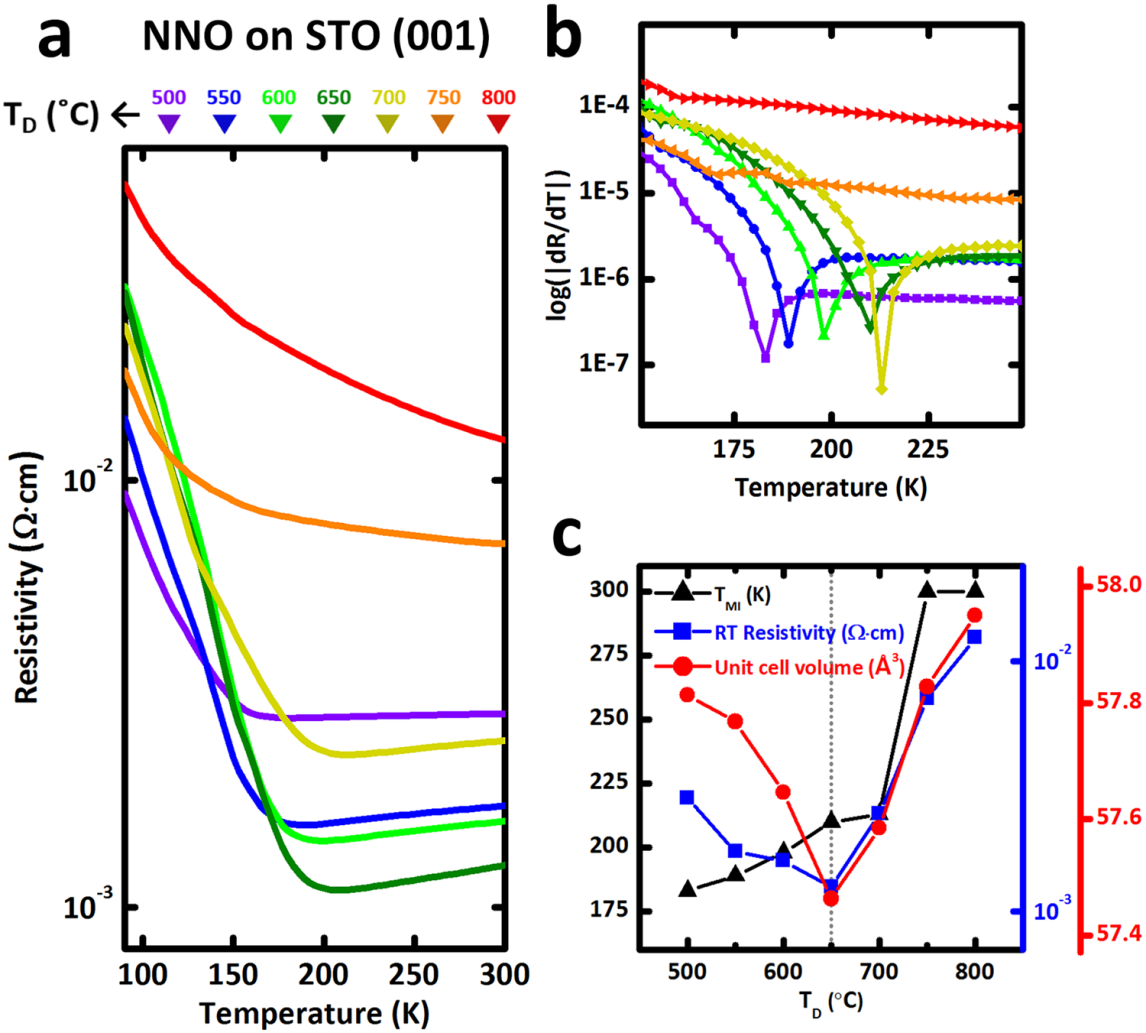
Correlation between MIT characteristics and structural modulation in oxygen-deficient NdNiO_3−δ_ films on STO substrates. (**a**) Temperature (*T*)-dependent in-plane resistivity for the NNO films on STO grown at various *T*_D_. (**b**) The first derivative of resistivity with respect to temperature (d*R*/d*T*) as a function of growth temperature (*T*_D_) to clarify *T*_MI_. (**c**) Correlation between *T*_MI_, room temperature (RT) resistivity and unit-cell volume in oxygen-deficient NdNiO_3−δ_ films. In case of NNO/STO grown at 750 and 800 °C, temperature dependence of resistivity curves show all insulating behaviors within the measured range (90 to 300 K), which indicates that *T*_MI_ is outside the plot window (*T*_MI_ > 300 K). Unlike NNO/LSAT, *T*_MI_ variation at 500 ≤ *T*_D_ ≤ 650 °C does not correspond to the unit-cell volume change in NNO/STO.

To probe how MIT characteristics can be modulated by adjusting δ in NdNiO_3−δ_ films, we measured temperature-dependent in-plane electrical resistivity for the NNO thin films on LSAT (Fig. 3a) and on STO (Fig. 4a) with different *T*_D_. All samples showed a sharp MIT, except grown at *T*_D_ = 750 and 800 °C, which shows degraded MIT characteristic. At room temperature (RT), the resistivity was higher in the optimized NNO on STO (>10^−3^ Ω cm at *T*_D_ = 650 °C) than in the optimized NNO on LSAT (<10^−3^ Ω cm at *T*_D_ = 600 °C); this difference indirectly confirms that the number of oxygen vacancies created to accommodate tensile strain in films increases as the amount of applied strain increases^9, 22–24, 29^. *T*_MI_ can be seen clearly in a plot of the first derivative of resistivity with respect to temperature (d*R*/d*T*) as a function of growth temperature (*T*_D_) (Figs 3b and 4b). Interestingly, the *T*_MI_ and RT resistivity were quite correlated with unit-cell volume in NNO films grown on LSAT: both quantities were lowest at the minimum of unit-cell volume (Fig. 3c); this result indicates that the electrical properties of this correlated oxide are strongly influenced by the degree of oxygen deficiency (δ). This tendency is also true for NNO/STO samples: the RT resistivity was lowest at the minimum of unit-cell volume, although *T*_MI_ variation from *T*_D_ = 500 to 650 °C does not correspond to the unit-cell volume change in NNO/STO.

## Discussion

**Figure 5:**
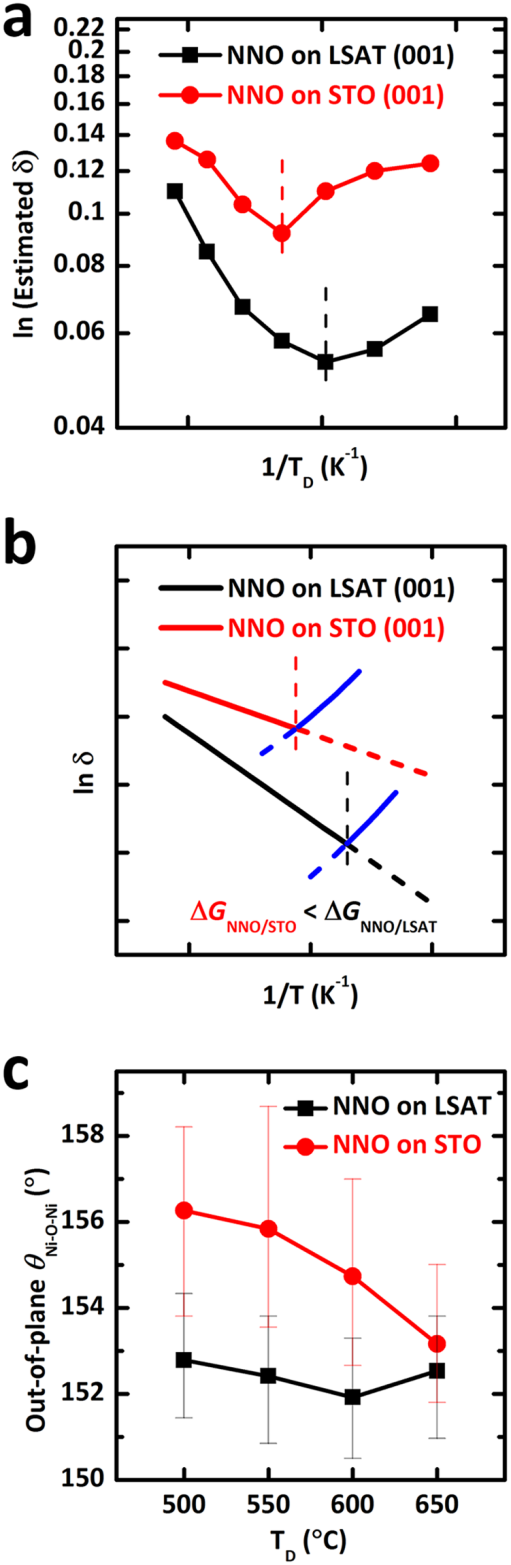
Quantitative estimation of oxygen deficiency (δ) and octahedral rotation in oxygen-deficient NdNiO_3−δ_ films. (**a**) Estimation of the oxygen deficiency (δ) in NdNiO_3−δ_ films using quantitative model. (**b**) Thermodynamic and kinetic model of inverse temperature (1/*T*)-dependent log δ for NNO/LSAT and NNO/STO to explain quantitative estimation of δ. (**c**) Out-of-plane Ni-O-Ni bond angles (θ_Ni-O-Ni_) with respect to *T*_D_. The Ni-O-Ni bond angle is calculated from θ_Ni-O-Ni_ = 2 arcsin(c/2d_Ni-O_), where Ni-O bond length (d_Ni-O_) was assumed to be equal to that of bulk NNO (1.942 Å). Note that there exists drastic change of Ni-O-Ni bond angle in *T*_D_ = 500 to 650 °C of NNO/STO.

The results of this experiment suggest that oxygen vacancies induced in NNO film increases as *T*_D_ deviates from the optimal *T*_D_ (600 °C for NNO/LSAT, 650 °C for NNO/STO). Oxygen vacancies increase the unit-cell volume of *R*NiO_3_^13, 20^, and also increase the RT resistivity of the metallic state^6, 7, 13–15, 32^. The degree of oxygen deficiencies in our NNO films was quantitatively estimated by introducing a simple model in order to analyze the relationship between unit-cell volume and oxygen vacancies in Fig. 5a. According to the empirical model, the unit-cell volume V of perovskite NdNiO_3−δ_ can be expressed as

2 $${\rm{V}}={A}^{3}{({r}_{B}+{r}_{anion})}^{3}$$

where *r*_*B*_ and *r*_*anion*_ are the ionic radii of the B-site cation (nickel site) and anion, respectively, and A is a constant close to 2.

By assuming oxygen-deficient NdNiO_3−δ_ due to the unchanged cation ratio (Nd/Ni) characterized by EDS (Fig. 1d), the effective cation and anion radii could be defined as

3 $${r}_{B}=(1-2\delta ){r}_{N{i}^{3+}}+(2\delta ){r}_{N{i}^{2+}}$$

4 $${r}_{anion}=(\frac{3-\delta }{3}){r}_{O}+(\frac{\delta }{3}){r}_{V}$$

where ${r}_{N{i}^{3+}}$, ${r}_{N{i}^{2+}}$, *r*_*O*_, and *r*_*V*_ are the ionic radii of Ni^3+^, Ni^2+^, oxygen, and oxygen vacancy, respectively^27, 33^. Consequently, the degree of oxygen deficiency (δ) (Fig. 5a) was roughly estimated to be ~0.05 and ~0.09 for NNO/LSAT grown at 600 °C and NNO/STO grown at 650 °C, respectively, from the measured unit-cell volume (Fig. 2b,d) using this empirical model. This consistent trend on oxygen deficiency in NNO films grown on two different substrates, i.e., the volcano shape as a function of *T*_*D*_, indicates that there should be different mechanisms to generate oxygen vacancies at high temperature (600 °C~800 °C for NNO/LSAT and 650 °C~800 °C for NNO/STO) and at low temperature (500 °C~600 °C for NNO/LSAT and 500 °C~650 °C for NNO/STO).

In case of high-temperature regime, a thermodynamic model of oxygen deficiency can be utilized to explain the mechanism based on given experimental evidences. The formation energy of oxygen vacancies (Δ*H*) decreases with the tensile strain^23^ and the degree of oxygen deficiencies (δ) can be expressed as follows:

5 $$\delta \propto exp(-\frac{{\rm{\Delta }}G}{kT})$$

where Δ*G* = Δ*H* − *T*Δ*S* (Δ*H* is the enthalpy of oxygen vacancy formation). If we ignore small entropy effects (Δ*G* = Δ*H*), the inverse temperature (1/*T*)-dependent log δ for NNO/LSAT and NNO/STO are estimated as shown in Fig. 5b. Moreover, as tensile strain increases, i.e., NNO/STO, the formation energy of oxygen vacancies decreases^23^; this trend can affect oxygen vacancies under various growth temperature (*T*_D_), and the change of oxygen deficiency causes systematic variation in *T*_MI_ as a function of *T*_D_. While this thermodynamic model provides a plausible explanation for the increase of oxygen deficiency in the high temperature regime (black and red solid lines in Fig. 5b), it does not account for the increase of oxygen deficiency in the low temperature regime.

In case of low-temperature regime, the insufficient mobility of oxygen in surface migration during the growth might lead to more oxygen-deficient NNO films on both substrates at lower temperature: Since oxygen atoms are unlikely to be supplied sufficiently at low temperature, the amount of oxygen incorporated into the thin film is kinetically limited, resulting in the increase of oxygen deficiency (blue solid lines in Fig. 5b) with decreasing the growth temperature. Considering higher concentration of oxygen vacancies in NNO/STO at low temperature, surface migration of oxygen was more suppressed in NNO/STO than in NNO/LSAT. Indeed, it was previously reported that the oxygen vacancy migration barrier of NNO/STO is larger than that of NNO/LSAT due to the tensile strain-induced suppression of oxygen vacancy migration to the surface^31^.

Despite the close relationship between unit-cell volume and *T*_MI_ above, the modulation of *T*_MI_ by unit-cell volume was inconsistent at 500 ≤ *T*_D_ ≤ 650 °C in highly tensile-strained NNO films on STO substrates. Beyond the explanation using unit-cell volume caused by oxygen vacancies, this can be explained by the influence of the octahedron distortion by tensile strain. In case of LaNiO_3_, in-plane tensile strain weakly modifies the out-of-plane Ni-O distance *d*_Ni-O_, but the out-of-plane Ni-O-Ni bond angle *θ*_Ni-O-Ni_ is highly sensitive to the strain^34^. Since our samples are all fully-strained state (RSM data, see Figs 2 and S1), it is reasonable to assume that in-plane Ni-O-Ni bond angle is fixed. Because *d*_Ni-O_ (out-of-plane) is almost insensitive to the strain, *θ*_Ni-O-Ni_ (out-of-plane) can be estimated from the relationship *θ*_Ni-O-Ni_ = 2arcsin(c/(2d_Ni-O_)) based on an assumption that *d*_Ni-O_ (out-of-plane) is equal to that of bulk NNO (1.942 Å). By inserting the observed out-of-plane lattice parameters (c), Ni-O-Ni bond angles (*θ*_Ni-O-Ni_) was estimated as shown in Fig. 5c. In NNO/STO, Ni-O-Ni bond angles drastically increased up to 156° as *T*_D_ deviated from the optimal *T*_D_~650 °C down to 500 °C; this trend is unlike that of NNO on LSAT. Because Ni-O-Ni bond angle determines the degree of overlap between Ni 3*d* and O 2*p* orbitals, orbital overlap increases and, therefore *T*_MI_ decreases despite the increase in unit-cell volume. Therefore, in highly tensile-strained NNO films on STO, the effect of octahedron distortion on *T*_MI_ is more influential than that of oxygen vacancies on *T*_MI_ in the range of *T*_D_ = 500 to 650 °C.

## Conclusion

In summary, we report systematic control of metal-insulator transition (MIT) in tensile-strained NdNiO_3−δ_ (NNO) epitaxial thin films by varying the degree of oxygen deficiency δ naturally by applying in-plane tensile strain, and by adjusting δ at growth temperature *T*_D_ through thermodynamic and/or kinetic control of defect formation. Unit-cell volume and MIT temperature *T*_MI_ both increased as δ increased. Our study provides useful approaches to finely modulate δ in correlated oxides, and provides insights on functional defects in rare-earth nickelate system.

### Experimental Methods

Epitaxial NdNiO_3_ (NNO) thin films were grown on (001)-oriented (LaAlO_3_)_0.3_-(SrAl_0.5_Ta_0.5_O_3_)_0.7_ (LSAT) and SrTiO_3_ (STO) single-crystal substrates (CrysTec Gmbh, Germany) by pulsed laser deposition with a KrF excimer laser (λ = 248 nm, Coherent Compex Pro 102 F). A polycrystalline stoichiometric NdNiO_3_ ceramic target was used to fabricate the thin film. During deposition, the laser pulse frequency was 10 Hz, laser energy was 2.2 J/cm^2^, and *p*(O_2_) was 300 mTorr. The growth ( = substrate) temperature *T*_D_ was varied from 500 to 800 °C. After each deposition, the sample was cooled to room temperature for 30 min under *p*(O_2_) = 300 mTorr without any *in-situ* post-annealing.

Structural characteristics of the NNO films were measured using θ–2θ scans and reciprocal space mapping (RSM) with a high-resolution X-ray diffractometer (XRD, Bruker D8 Discover X-ray diffractometer) with Cu K_α1_ radiation (λ = 1.5406 Å). Cation stoichiometry of the film was determined in high vacuum by using a field-emission scanning electron microscope (FE-SEM, JEOL JSM-7401F) coupled with an energy dispersive spectroscopy system (EDS, Oxford INCA). In-plane electrical transport was measured using the Van der Pauw method at 90 ≤ *T* ≤ 300 K during heating.

To check time-dependent change of oxygen deficiency in NNO films, we re-examined the change in the XRD peaks of all NdNiO_3−δ_ samples used for the manuscript after 10 months. All samples were stored in the desiccator at room temperature for 10 months without any capping layer.

## Electronic supplementary material

SI
