# Supplementary material for: Influence of tensile-strain-induced oxygen deficiency on metal-insulator transitions in NdNiO3−δ epitaxial thin films
Source: Sci Rep. 2017 Jul 5;7:4681. doi: 10.1038/s41598-017-04884-2 (PMC5498495; doi:10.1038/s41598-017-04884-2)
Supplement: Supplementary file 1 — SI [file 41598_2017_4884_MOESM1_ESM.pdf]

Supplementary information for

Influence of tensile-strain-induced oxygen deficiency on metal-insulator transitions in $NdNiO_{3-\delta}$ epitaxial thin films

Seungyang Heo[1)], Chadol Oh[2)], Junwoo Son[2), *], Hyun Myung Jang[1, 2)]

[1)] Division of Advanced Materials Science (AMS), Pohang University of Science and Technology (POSTECH), Pohang 790-784, Republic of Korea

[2)] Department of Materials Science and Engineering (MSE), Pohang University of Science and Technology (POSTECH), Pohang 790-784, Republic of Korea

* jwson@postech.ac.kr

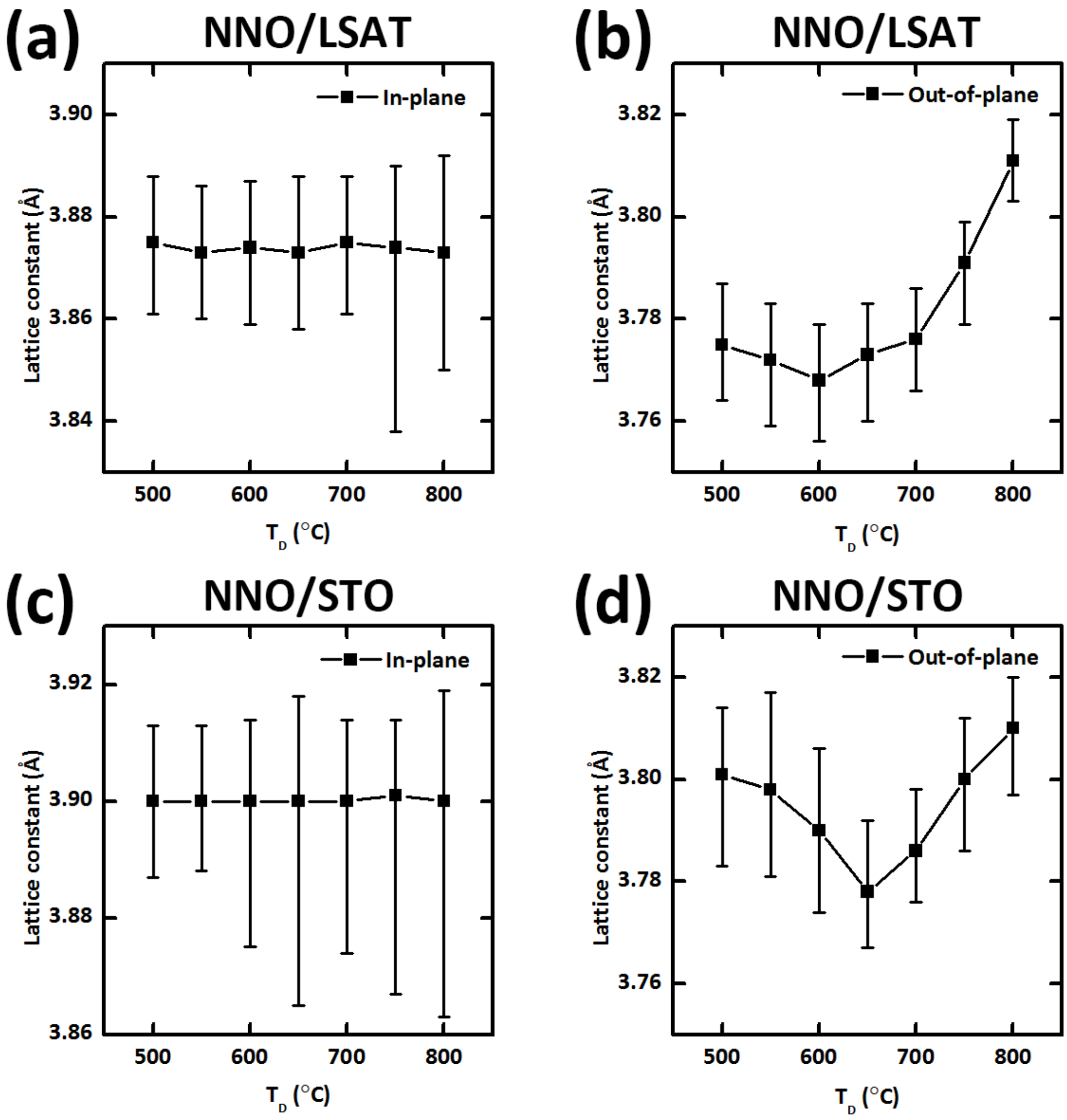

Figure S1 | Lattice constants of NNO/LSAT and NNO/STO. (a) Extracted in-plane lattice constants of NNO thin films grown at various T_D from RSM measurement. This data shows that all NNO thin films are fully-strained on LSAT substrate. (b) Extracted out-of-plane lattice constants of NNO thin films grown on LSAT. (c) Extracted in-plane lattice constants of NNO thin films grown at various T_D. This data shows that all NNO thin films are fully-strained on STO substrate. (d) Extracted out-of-plane lattice constants of NNO thin films grown on STO.

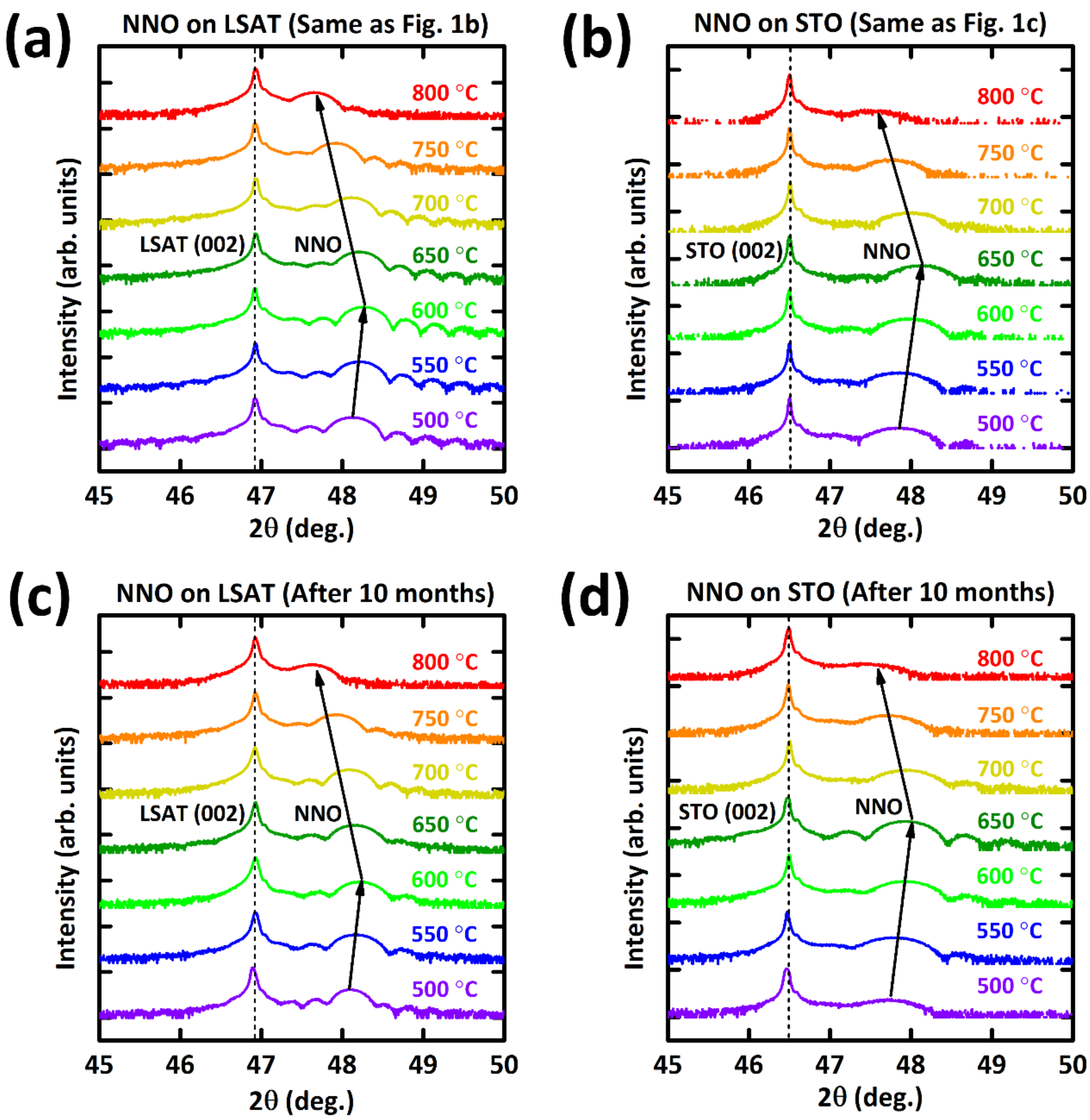

Figure S2 | Stability of oxygen-deficient $NdNiO_{3-\delta}$ thin films under ambient condition. (a), (b) XRD θ-2θ scan of NNO epitaxial thin films on (001) LSAT and (001) STO as a function of growth temperature (T_D) (Same as **Fig. 1b** and **Fig. 1c**, respectively). (c), (d) XRD θ-2θ scan of NNO epitaxial thin films after 10 months. Not only did all samples maintain their XRD pattern, but the trend of volcano shapes was also unchanged even after 10 months.
